# 

**Published:** 1865-07

**Authors:** 


### BOOKS AND PAMPHLETS RECEIVED

*The Annual Announcement of the St. Louis Medical College, Session* 1865–6, *and Catalogue for* 1864–5. St. Louis: GEORGE KNAPP & Co, 1865.

*Sixth Annual Announcement of the Miami Medical College of Cincinnati.* Cincinnati: A. MOORE, 1865.

*On Sleep and Insomnia.* BY WILLIAM A. HAMMOND, M. D., *of New York.* 1865.

*Hand-Book of Skin Diseases for Students and Practitioners.* BY THOMAS HILLIER, M. D., *London.* Philadelphia: BLANCHARD & LEA, 1865.

*Thirty-Third Annual Annoucement of the Faculty of Medicine of the McGill University. Session* 1865–6. *Montreal.*

*Annual Circular of the Bellevue Hospital Medical College, City of New York, for* 1865–6.

DELAY IN APPEARANCE OF JOURNAL.—By an accident in the office of publication, our journal for this month was "*pied*" instead of published, which accounts for the late appearance.

### INDEX.

Page.

**A**

Abstract of the Proceedings of the Buffalo Medical Society, 44, 86, 132, 188, 336, 381, 414, 452, 502, 549.
Abstract of the Proceedings of the Medical Society of South Western New York... 505
Acid, Carbolic ........................... 25
Acid, Chromic ........................... 496
Acid, Hydrocyanic in Mania ............... 152
American Medical Times, suspended....... 80
American Medical Association and Dr. Morton ............................ 150
American Medical Association, Transactions of ............................476, 593
American Medical Association.....480, 518, 560
American Dental Association, Officers of... 61
America, Medical Fees in................. 392
Aneurism, Subclavian, Successful Operation—By A. W. Smith, M. D........... 346
Aneurism of the Aorta.................216, 393
Aneurism, Popliteal ...................... 3[illegible]5
Address—By S. Hart, M. D................. 125
Address—By Dr. Bigelow ................. 560
Address—By Prof. Wm. D. Wilson......... 231
Addison's Disease—By J. F. Miner, M. D....590
Amputation of Shoulder Joint—By J. F, Miner, M. D.......................... 378
Amputation of Leg........................ 393
Amputation of Leg—By J. F. Miner, M. D.. 421
Annual Address before Erie County Medical Society—By S. Wetmore, M. D..... 368
Annual Report of the Provost Marshal General ................................. 563
Annual Meeting of the New York State Medical Society ....................... 386
Annual Address before the Medical Society of the County of Kings—By S. Hart, M. D................................. 125
Annals of the Albany County Medical Society ................................. 206
Anomalous Cases of Parturition and Gestation ................................ 523
Alphabetical Index to Braithwaite's Retrospect ............................. 520
Asylum, Insane, Advisory Medical Board for .................................. 135
Ankle, Resection of ...................... 211
Ankle Joint, Resection of—By Wm. Canniff, M. D., M. R. C. S. Eng. .......... 463
Autopsies, Fees for making................ 155
Apoplexy, Cerebral........................ 351
Absence of Epiglottis..................... 353
Army Surgeon's Manual—By Wm. Grace, 346, 612
Apothecaries and the Medical Profession... 398
Anæsthesia Produced by Chloroform....... 473
Assassination of President Lincoln......... 474
Anus, Imperforate........................ 522
Atalectasis .............................. 530

**B**

Buffalo General Hospital, Report of........ 11
Buffalo General Hospital, Inspection of .... 79
Buffalo Medical Association, Abstract of the Proceedings of, 44, 86, 132, 188, 336, 381, 414, 452, 502, 549.
Buffalo Medical College, Lectures in....... 120
Buffalo Medical College Commencement... 428
Books Received, 39, 79, 120, 164, 206, 347, 3[illegible]1, 436, 479, 520, 567, 613.
Brown—Sequard .......................... 167
Berkshire Medical College Commencement. 208
Bromide of Potassum...................... 31
Bergamot, Instantaneous Cure of Itch...... 154
Brain, Tuberculous Tumor of.............. 210

Page.

Brain, Inflammation of .................. 410
Bubo, Sloughing.......................... 483
Black Vomit.............................. 354
Bone, Remarkable Reproduction of—By J. F. Miner, M. D........................ 378
Bone from Wound, Speculæ of............. 397
Beasley's Book of Prescriptions ........... 309
Birth, Extraordinary...................... 392
Bowels, Ulceration of .................... 123
Breast, Tumor of......................... 400
Biographical Sketch of Living Physicians... 425
Bacteridian and Malignant Pustules........ 466
Braithwaite's Retrospect, Index to ........ 520
Baker Brown's Operation for Lacerated Perineum ............................ 530
Bigelow, Dr., Address by ................. 560
Bennet, J. H. M. D., Hypodermic Treatment of Uterine Pains ...................... 101
Buttles, M. S., M. D., Sea-Tangle (Laminaria Digitata) ........................ 187
Barrett, S., M. D., Delirium Tremens...... 339
Bell, Dr. A. N., Paper on Disinfection..... 437
Baron, Liebig............................ 392
Bissell, D. P., M. D., Address............ 613

**C**

Card, Dr. Hammond ....................... 75
Cancers.................................. 579
Cancer of the Lip......................... 497
Cancer, Medullary........................ 121
Calculus, Vesico Renal.................... 408
Cataract, Operation for................... 62
Caries ................................... 582
Calcareous Tumor......................... 394
Catarrh—Inhalation....................... 406
Chronic Inflammation and Displacement of Uterus ................................ 76
Chronic Diarrhœa—By Robt. Taylor, M. D. 8
Charges against Brigadier General William A. Hammond .......................... 28
Chloroform, Preservation of.............. 38
Chloroform, Death from .................. 21
Chloroform, Poisoning by ................ 124
Cheapest Disinfectant.................... 72
Change of the Color of the Hair—By J. F. Miner, M. D.......................... 93
Chorea, Turpentine in.................... 470
Chapman, John, M. D., M. R. C. P., New Method of Treating Disease.......... 54
Climatic Distemperature.................. 71
Clinic for the Diseases of Females........ 106
Clinical Remarks on Surgical Cases—By J. F. Miner, M. D.................194, 378, 421
Club-Foot, Causes of ..................... 226
Closure of a Vesico-Vaginal Fistula—By J. R. Lothrop, M. D..................... 457
Compound Dislocation of Tibia............ 499
Cow-Pox, Origin of, and Nature of Vaccine Virus ................................ 70
Comprehensive Dictionary—By J. Thomas, M. D.................................. 163
Crystaline Constituents of Plants ......... 150
Cerebral Apoplexy ....................... 351
Cerebro-Spinal Meningitis .............367, 571
Colic .................................... 495
Confederate Prisons ..................... 426
Cellulitis, Pelvic, Mercurials in ......... 472
Cystitis ................................. 581
Cystic Disease of the Ovary............... 125
Cystic Tumor of the Breast............400, 581
Condyloma ............................... 533
Convulsions ......................489, 521, 528
Cyclones and Sickness in India............ 557
Conkling, Dr. J. T., Black Vomit......... 354

Page.
Committee of the American Medical Association ... 207
Catalepsy...e ... 580

D

Davis, D. N. S, Address ... 593
Deaths in the City of Buffalo, Report of, 40, 80, 167, 168, 208, 348, 568.
Diarrhœa, Chronic ... 8
Diphtheritic Paralysis ... 6
Diphtheria ... 218, 482
Diphtheria, Spurious ... 104
Diphtheria, Tracheotomy in ... 169
Diphtheria—By Slade ... 342
Diphtheria, with Scarlet Fever ... 484
Diphtheria, Inhalation in ... 531
Diagnosis, Medical—By J. M. Da Costa, M. D. ... 36
Diagnosis, Surgical—By G. H. B. McLeod, M. D. ... 200
Disinfection—By A. N. Bell, M. D ... 437
Delirium Tremens ... 339
Diary and Book of Engagements ... 163
Disease, New Method of Treating ... 54
Disease of the Kidney, Granular ... 405
Displacement of Uterus, and Chronic Inflammation of ... 76
Dunglison's Medical Dictionary ... 478
Dropsy ... 486
Dysmenorrhœa ... 494
Dislocation of Tibia, Compound ... 499
Dislocation of Wrist ... 393
Deformities of the Feet, and Treatment of. 221
Druggist's Receipt Book ... 520
Development of Phthisis, Influence of Pneumonia in ... 155
Depression of Skull with Fracture ... 209, 397
Dismissal of Surgeon General William A. Hammond, a Statement of the Causes .. 485
Diabetis Millitos ... 580

E

Effects of the Excessive Use of Sugar on the System ... 147
Erie County Medical Society, Meeting of ... 341
Erie County Medical Society, Representation in the New York State Medical Society ... 197
Erie County Medical Society, Annual Address—By S. W. Wetmore, M. D ... 368
Erie County Medical Society, Semi-Annual Meeting ... 568
Epiglottis. Absence of ... 349
Epilepsy Trephining in — By J. F. Miner, M. D ... 421
Entozoa, Specimen of ... 399
Encysted Calculus ... 490
Excision of the Radius—By J. F. Miner, M. D. ... 378
Epidemic of Typhus, Typhoid and Spotted Fever ... 510
Epidemic of Plague in St. Petersburgh, Russia ... 555
Epidemic Erysipelas—By Thos. D. Strong M. D ... 507
Enos, Dr. D. C., on Deformities of the Feet, and their Treatment ... 221
Enos, Dr. D. C., Absence of the Epiglottis. 353
Explanation ... 568
Extirpation of Melanoid Tumor—By J. F. Miner, M. D ... 421
Ephemeral Fever ... 486
Epithelioma ... 569

F

Fracture of the Skull ... 209, 397
Fracture of the Liver ... 402
Fracture of the Neck of the Femur ... 481
Fracture of the Tibia ... 499
Fever, Spotted, Notice ... 207
Fever, Treatment of, by Subcutaneous Injection of Quinia ... 392
Fever, Scarlet, with Diphtheria ... 484
Foreign Bodies in Meatus Auditorius—By J. M. Foster, M. D ... 41
Foreign Iodide of Potassium, Purity of ... 149
Fatty Heart, Smoking as a Cause ... 145
Fatty Degeneration of the Heart and Kidneys ... 532
Federal Army, Surgeon General of ... 470
Foot, Cause of Club ... 226
Femoral Hernia, Operation for ... 485
Functions and Disorders of the Reproductive Organs of Childhood ... 478
Fistula, Vesico-Vaginal, Operation for—By J. R. Lothrop, M. D ... 457
Fibro-Cartilaginous Tumor, Case of ... 189
Foster, Jerome M., M. D., Foreign Bodies in Meatus Auditorius ... 41
Flexions of the Uterus, Sea-Tangle used in. 187
Fuller on Rheumatism ... 37

G

Gross, Surgery ... 213
Garrod, Dr., on Bromide of Potassium ... 31
Goff, Dr. H. B., Paper on an Anitomical Specimen ... 381
Gould, William, M. D., Fibro-Cartilaginous Tumor ... 189
Gilfillan, Dr. Wm , Tracheotomy in Diphtheria ... 169
Gay, Dr., Paper on Erysipelas ... 86
Geneva Medical College, Address to Graduating Class ... 231
Gonorrhœa and Syphilis, Treatise on—By S. Durkee, M. D. ... 159
Gun-Shot Wound of the Thigh ... 493
Gun-Shot Wound, Amputation at Shoulder Joint—By J. F. Miner, M. D ... 378
Glaucoma—By P. D. Keyser ... 391
Glaucoma, Iridectomy in ... 559
Gastritis, Acute ... 495
Glycerine and its Uses ... 520
Granular Disease of the Kidneys ... 405

H

Hammond, Brigadier General William A., Charges against ... 28
Hammond, Late Surgeon General ... 73
Hammond, Military, Medical and Surgical Essays ... 115
Hammond, Lectures on Venerial Diseases .. 204
Hammond, Causes of Dismissal ... 435
Hypertrophy of the Heart ... 400
Hypodermic Treatment of Uterine Pains ... 101
Hydrothorax ... 121
Hemorrhoids, Internal, Chromic Acid used in ... 496
Hemorrhage from the Bowels ... 492
Hemorrhage, Umbilical ... 366
Heart and Kidneys, Fatty Degeneration of .. 532
Heart, Action of ... 473
Honor to the Brave ... 165
Hygene and Military Surgery—By Frank H. Hamilton, M. D ... 329
Hematuræ ... 488
Hæmoptysis ... 485
Hernia, Operation for ... 485, 497, 582
Hydrocele, Treatment of ... 552
Hysteria ... 489
Hart, Samuel, M. D., Annual Address ... 125
Hutchinson, Dr., Compound Dislocation of Tibia ... 499
Homberger, Julius, M. D , Standard Operation for Cataract ... 62
Hassall, A. H., M. D., on Tincture of the Perchloride of Iron ... 553
Household Poems ... 6.3

Page.

I

Inflammation of Base of the Brain. ....... 410
Illinois State Medical Society, Meeting of... 567
India, Small-Pox in .................. 518
Influence of Plurisy in Development of Phthisis ........ ...................... 155
Ilium, Rupture of ......................... 217
Illustrated Magazine...................... 166
Iridectomy in Glancoma.................... 559
Iron, Tincture of the Perchloride of........ 553
Iron, Preparation of an Improved Wine of.. 149
Infant Therapeutics, Essays on—By J. B. Beck, M. D............................ 119
Infanticide in London ...................... 167
Injection of Quinia in Treatment of Malarious Fevers........ .................. 392
Indiana State Medical Society, Transactions of .............. ................... 120
Inspector's Report of New York City... ... 611
Intra-Pericardial Cyst ..................... 536
Inhalation in Diphtheria .................. 531
Inhalation of Chloroform.................. 473
Imperforate Anus......................... 522
Identification of Deceased Soldiers......... 151
Itch, Instantaneous Cure by Bergamot..... 154
Innominata, Successful Legation of........ 156
Income Tax by Physicians.................. 158
Iodide of Potassium, Purity of Foreign..... 149
Iritis, Mercurials in...................... 472
Influenza ................................ 486
Ileus...................................... 584

J

Journal, New York Medical ............... 479
Johnson, Dr. John G., Resection of Ankle. 211
Jollie, Dr. William, Treatment of Hydrocele ................................. 552

K

Kings County Medical Society, Transactions of..121, 158, 169, 209, 349, 393, 437, 481, 521, 569
Kidneys, Granular Disease of.............. 405
Kidneys, Fatty Degeneration of............ 532
Keyser, P. D., Glancoma ................. 391
Knee-Joint, Injury to—By J. R. Lothrop, M. D.................................. 585

L

Lectures on Orthopœdic Surgery........... 77
Lectures on Medical Education ............ 78
Lectures on Venerial Diseases—By Hammond............................... 204
Lectures on Surgical Pathology—By James Paget, F. R. S......................... 519
Late Surgeon General Hammond.......... 73
Letters from Dr. S D. Willard ........... 197
Lee, Professor, Letters from.... ........197, 340
Laryngoscopal Medication................. 344
Literary Journals.......................... 820
Liebig, Baron............................. 392
Local Experience......................... 528
Lacerated Perineum, Baker Brown's Operation for.................................. 530
Lexicon, Medical.....................478, 566
Lothrop, J. R., M. D., Closure of Vesico-Vaginal Fistula....................... 457
Lothrop, J. R., M. D—Injury to Knee-Joint. 585
Lockwood, Dr., Simple and Specific Gonorrhœa................................. 417
Leg, Spasm of, after Labor ............... 494

M

Medical Society of the State of New York, 161, 386.
Medical Society of the County of Kings, Transactions of, 121, 158, 169, 209, 349, 393, 437, 481, 521 569.
Medical Society of the State of Ohio........ 205
Medical Society of Erie County....341, 363, 568

Page.

Medical Society of the State of Pennsylvania ..............................566, 347
Medical Society of the Southwestern New York ................................... 5 5
Medical Society of the State of Illinois..... 567
Miner, J. F., M. D., Exfoliation of Bone.... 453
Miner, J. F., M, D., Specimen of Bone from the Upper Part of Tibia ........... ... 414
Miner, J. F., M. D., Exsection of Radius.... 378
Miner, J. F., M. D , Gun-Shot Wound, Amputation of Shoulder Joint............. 378
Miner, J. F., M. D., Amputation of Leg.... 421
Miner, J. F., M. D , Surgical Diseases of Women.... .....................1, 48, 81
Miner, J. F., M. D., Uterine Polypi ........ 1
Miner, J. F., M. D., Trephining in Epilepsy .................................. 421
Miner, J. F., M. D., Ovarion Tumor........ 48
Miner, J. F., M. D., Extirpation of Melanoid Tumor................................ 421
Miner, J. F., M. D., Rupture of the Perineum ........................ ...... 81
Miner, J. F., M. D., Change of the Color of the Hair, Case of..................... 93
Miner, J. F., M. D., Clinical Remarks on Surgical Cases................194, 378, 421
Miner, J. F., M. D., Necrosis of Tibia, with Contraction of Tendo-Achillis.......... 194
Miner, J. F., M. D., Necrosis after Amputation, Remarkable Reproduction of Bone, 378
Miner, J. F., M. D., Schirrhus Tumor...... 194
Miner, J. F., M. D., Addison's Disease ..... 590
Medical College, Buffalo, Lectures in....... 120
Medical College, Buffalo, Commencement.. 428
Medical Diagnosis—By J. M. Da Costa, M. D.................................. 36
Medical News Items...................... 39
Medical Men, their Relation to each Other and the Public ....................... 111
Medical Men as Coroners ................. 156
Medical Profession and Apothecaries....... 388
Meachem, J. G., M. D., Diphtheritic Paralysis... ................................ 6
Morton, Dr. James, Abuse of Caustics...... 21
Melanoid Tumor, Extirpation of............ 421
Morphia, Anæsthesia kept up by Subcutaneous Injections of .................. 473
Mercurials in Pelvic Cellulitis and Iritis.... 472
Military Surgery and Hygiene—By Frank H. Hamilton, M. D..................... 389
Man and His Relations—By S. B. Brittan, M. D.................................. 345
Materia Medica and Therapeutics Stelle.... 202
Military, Medical and Surgical Essays—By Hammond .......... ............... 115
Meatus Auditorius, Foreign Bodies in—By J. M. Foster, M. D.................. 41
Manual of the Practice of Medicine—By Thos. H. Tanner, M. D., F. L. S. ....... 38

N

Necrosis ................................. 484
Necrosis, after Amputation—By J. F. Miner, M. D................................. 378
Necrosis of Tibia, with Contraction of Tendo-Achillis—By J. F. Miner. M D... 198
Notice of Spotted Fever ....... .......... 208
Nostrils, Removal of Foreign Bodies from.. 407
Nephralgia ............................ 484
Noggerath's, Clinic for Diseases of Females. 106
Nealton, M........................... ... 167

O

Operation for Strabismus made easy........ 559
Operation for Cataract—By J. Homberger, M. D................................ 62
Operation for Hernia........ ..........485, 497
Operation for Subclavian Aneurism—By A. W. Smith, M. D....... ................ 346

Page.
Operation for Lacerated Perineum, Baker Brown's.......... 530
Orthopœdic Surgery, Lectures on.......... 77
Opthalmiscope, A Simple.......... 151
Ovarion Cyst and Vagnial Stricture—By W. Gould, M. D.......... 189
Ovarion Tumor—By J. F. Miner, M. D..... 48
Ovary, Cystic Disease of.......... 125
Ohio State Medical Society.......... 205
Oil of Tansy, Poisoning by.......... 526

P

Presentation.......... 207
Physiciau's Income Tax.......... 158
Physician's Dose and Symptom Book...... 163
Physician's Visiting List Diary and Book of Engagements.......... 163
Physician's Prescription Book.......... 613
Paralysis.......... 521
Puerperal Convulsions..........489, 521, 528
Paracentesis Thorasis.......... 121
Preparation of an Improved Wine of Iron.. 149
Perforation of the Stomach.......... 219
Petitiou of the late Surgeon-General to the Senate of the United States.......... 347
Petroleum.......... 392
Petroleum in Scabies.......... 472
Pregnancy, Spurious.......... 537
Plague in Russia.......... 555
Proceedings of the American Medical Association.......... 560
Paget, James, F. R. S., Lectures on Surgical Pathology.......... 519
Pearce, J. F., M. D., Epidemic of Typhus, Typhoid and Spotted Fever.......... 510
Peters, Dr. J. A., Oleum Tanaceti in Abortion.......... 382
Pericarditis, Specimen of.......... 395
Phthisis, Development of.......... 155
Pneumonia in the Army of the Potomac.... 124
Pneumonia.......... 573
Pertussis.......... 487
Periostitis.......... 492
Pathological Specimen—By Dr. Speir...... 394
Popliteal Aneurism, Specimen of.......... 395
Pyæmia..........400, 412
Pharmaceutists and Druggists' Receipt Book 520
Ptyslism.......... 574

Q

Quack Advertisements, the Secular and Religious Press.......... 514
Quinia, Subcutaneous Injection of.......... 392

R

Rheumatism—By Wm. H. Fuller, M. D.... 36
Remedies, Report of New..........65, 94
Rupture of the Uterus.......... 349, 574
Relations of Medical Men to each other and the Public.......... 111
Representation of Erie County Medical Society in New York Medical Society..... 197
Representation of the University of Buffalo in New York Medical Society.......... 197
Rectum and Uterus, Scirrhus of.......... 352
Railroad, Killed and Wounded by.......... 392
Rochester, Prof. T. F., Empyæmia.......... 150
Rochester, Prof. T. F., Pelvic Hæmatocele. 452
Rochester, Prof. T. F., Spotted Fever, Cases of.......... 502

S

Surgical Pathology—By J. Paget, F. R. S... 519
Surgeon General of the Federal Army..... 470
Surgeon General, Late, Petition to the United States Senate.......... 347
Surgeon General Hammond, Late.......... 73
Surgeon General of New York.......... 392
Surgeon General Willard, Death of........ 475
Surgery, Gross, Treatise on.......... 213
Surgical Cases in Buffalo General Hospital—By J. F. Miner, M. D..........194, 378, 421
Struck Oil.......... 206
Sea-Tangle, Use of, in Flexions of the Uterus—By M. S. Buttles, M. D.......... 187
Stelle's Therapeutics.......... 302
Superinvolution.......... 106
Subinvolution.......... 106
Smoking as a Cause of Fatty Heart........ 145
Sugar, Effects of the Excessive use of, on the System.......... 147
Soldiers, Identification of Deceased.......... 151
Slade on Diphtheria.......... 342
Syphilis, Vaccination in.......... 473
Specula of Bone from Wound.......... 397
Small-Pox in India.......... 518
Spinal Meningitis.......... 523
Spurious Pregnancy.......... 537
Strabismus, Operation Made Easy.......... 559
Smith, G. S., Esq., on Spurious Diphtheria. 104
Strong, Thomas D., M. D., Report on Epidemic Erysipelas.......... 507
Sick and Wounded in Confederate Prisons.. 426
Scarlatina.......... 491
Spasm of the Leg after Labor.......... 494
Strangulated Hernia.......... 496

T

Transactions of the Medical Society of the State of Indiana.......... 120
Transactions of the Medical Society of the County of Kings, 121, 169, 209, 349, 393, 437, 481, 521, 569.
Transactions of the Medical Society of the State of New York.......... 161
Transactions of the Medical Society of the State of Pennsylvania..........347, 566
Treatment of Itch by Bergamot.......... 154
Treatment of Malarious Fevers.......... 392
Treatise on Gonorrhœa and Syphilis—By S. Durkee, M. D.......... 159
Tracheotomy in Diphtheria.......... 170
Tendo-Achillis, Operation for Contraction—By J. F. Miner, M. D.......... 194
Therapeutics and Materia Medica—Stelle... 209
Therapeutics.......... 408
Tincture of Digitalis used in Delirium Tremens.......... 339
Tuberculosis.......... 402
Tumor, Calcarious.......... 394
Tumor of Breast.......... 400
Tumor, Extirpation of—By J. F. Miner, M. D.......... 421
Tumor, Fibro-Cartilaginous.......... 189
Tumor, Scirrhus—By J. F. Miner, M. D..... 194
Turpentine in Chorea.......... 407
Trephining in Epilepsy—By J. F. Miner, M. D.......... 421
Thigh, Gun Shot Wound of.......... 493
Tibia, Fracture of.......... 499
Tibia, Compound Dislocation of.......... 499
Tibia, Necrosis of—By J. F. Miner, M. D... 194
Treatment of Hydrocele.......... 552
Tincture of the Per-Chloride of Iron....... 553

U

Ulceration of Bowels.......... 123
Uterine Polypi—By J. F. Miner, M. D...... 1
Uterine Pains, Hypodermic Treatment of.. 101
Uterine Therapeutics.......... 111
Umbilical Hemorrhage.......... 366
Ulceration of Larynx.......... 353
University of Buffalo, Representations in New York State Medical Society....... 197
Uræmia.......... 411
Uterus, Scirrhus of.......... 352
Uterus, Rupture of..........349, 574
Uterus, Chronic Inflammation and Displacement of.......... 76

Page.

**V**

Varicocele.............................. 523
Volume Four..........................33, 609
Visiting List, Diary and Book of Engagements.............................. 163
Vaginal Stricture.......................... 189
Venereal Diseases, Lectures on—By Dr. Wm. A. Hammond.................... 204
Vesico-Renal Calculus.................... 408
Vesico-Vaginal Fistula—By J. R. Lothrop, M. D.............................. 457
Vaccination and Re-vaccination............ 433

**W**

Watson, Dr. James, on Carbolic Acid...... 25

Page.

Women, Surgical Diseases of—By J. F. Miner, M. D......................1, 48, 81
Willard, S. D., M, D., Letters from......... 197
Willard, Surgeon General, Death of........ 475
Wrist Dislocation.......................... 393
Wilson, Prof. Wm., Address to the Gradua-Class of Geneva College.............. 231
Wetmore, Dr. S. W., Annual Address...... 368
White, Prof. J. P., Polypus of the Uterus... 336
White, Prof. J. P., Subinvolution and Super-involution............................ 91
Washburn, Dr. C. E., Resolutions on the Occasion of the Death................ 507

